# Carrier-free highly drug-loaded biomimetic nanosuspensions encapsulated by cancer cell membrane based on homology and active targeting for the treatment of glioma

**DOI:** 10.1016/j.bioactmat.2021.04.027

**Published:** 2021-05-01

**Authors:** Yueyue Fan, Yuexin Cui, Wenyan Hao, Mengyu Chen, Qianqian Liu, Yuli Wang, Meiyan Yang, Zhiping Li, Wei Gong, Shiyong Song, Yang Yang, Chunsheng Gao

**Affiliations:** a College of Pharmacy, Henan University, Kaifeng, 475000, PR China; b State Key Laboratory of Toxicology and Medical Countermeasures, Beijing Institute of Pharmacology and Toxicology, Beijing, 100850, PR China

**Keywords:** Nanosuspensions, Cancer cell membrane, Biomimetic drug-delivery systems, Gliomas, Blood-brain barrier, Paclitaxel

## Abstract

Nanosuspensions, as a new drug delivery system for insoluble drugs, are only composed of a drug and a small amount of stabilizer, which is dispersed in an aqueous solution with high drug-loading, small particle size, high dispersion, and large specific surface area. It can significantly improve the dissolution, bioavailability, and efficacy of insoluble drugs. In this study, paclitaxel nanosuspensions ((PTX)NS) were prepared by an ultrasonic precipitation method, with the characteristics of simple preparation and easy repetition. With the help of a homologous targeting mechanism, a kind of glioma C6 cancer cell membrane (CCM)-coated (PTX)NS was developed and modified with ^D^WSW peptide to obtain ^D^WSW-CCM-(PTX)NS with the functions of BBB penetration and tumor targeting. The results showed that the cancer cell membrane could effectively camouflage the nanosuspensions so that it was not cleared by the immune system and could cross the blood-brain-barrier (BBB) and selectively target tumor tissues. Cell uptake experiments and *in vivo* imaging confirmed that the uptake of ^D^WSW-CCM-(PTX)NS by tumor cells and the distribution in intracranial gliomas increased. Cytotoxicity test and *in vivo* anti-glioma studies showed that ^D^WSW-CCM-(PTX)NS could significantly inhibit the growth of glioma cells and significantly prolong the survival time of glioma-bearing mice. Finally, the cancer cell membrane coating endowed the nanosuspensions with the biological properties of homologous adhesion and immune escape. This study provides an integrated solution for improving the targeting of nanosuspensions and demonstrates the encouraging potential of biomimetic nanosuspensions applicable to tumor therapy.

## Highlights

• Paclitaxel nanosuspensions with high drug-loading and without carrier.
• Biomimetic nanosuspensions wrapped by peptide-modified cancer cell membranes.
• Penetrate BBB and BBTB to transport drugs to glioma.
• Dual effects of active and homology targeting improve therapeutic efficiency.

## Graphical abstract

**Figure:**
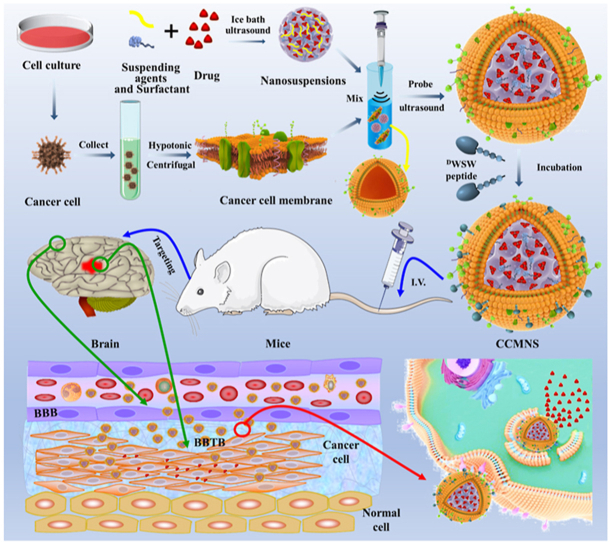

## 1 Introduction

In the process of new drug research and development, most of the candidate drugs are eliminated due to low solubility and bioavailability. The scope of application of many clinical drugs is also limited for these reasons. To overcome all these drawbacks, nanodrug delivery systems (NDDS), which include liposomes, niosomes, solid lipid nanoparticles, and nanoemulsions, have been widely used [1]. Among them, nanosuspensions (NS) with the characteristics of simple preparation and good repeatability are a new dosage form developed for insoluble drugs in recent years [2,3]. By using a small amount of surfactant or polymeric material as a stabilizer, the drug particle size is controlled at the nanometer level. This significantly increases the surface area, solubility, dissolution rate, and bioavailability of the drug to reduce the drug dosage and avoid adverse drug reactions caused by an overdose. Since there is no carrier, as “pure drug particles”, they have a high drug loading capacity of close to 100% [4,5].

The physical stability of nanosuspensions has always been the bottleneck of their application [6]. Drugs in a liquid environment can sediment and aggregate and display other unstable phenomena. In addition, drug nanoparticles are easily recognized and eliminated by the reticuloendothelial system or mononuclear phagocytes in the immune system after entering the blood. Moreover, non-specific proteins and biomolecules adhere easily to the surface of nanoparticles, which further interferes with the interaction between nanoparticles and biological systems. In tumor treatment, nanosuspensions usually achieve non-specific targeting under the enhanced permeability and retention (EPR) effect *in vivo*, and the targeting efficiency is low [7].

Therefore, after the preparation of a paclitaxel nanosuspension, this study tried to camouflage nanoparticles with new biomimetic materials to build a biomimetic drug-delivery systems (BDDSs) [8]. Among many biomimetic materials, cell membranes are materials that can endow the nanoparticles with unique biological properties. By fusing cell membranes onto the surface of the drug nanoparticles, the nanoparticles can have the properties of the original cell membrane, and the stability of the nanosuspensions is improved [[9], [10], [11]]. Among different cell membrane materials, cancer cell membranes have domains that adhere to homologous cells and homologous binding proteins, which can provide the nanoparticles coated with tumor cell membranes with tumor-targeting properties [[12], [13], [14], [15]].

**Fig. 1:**
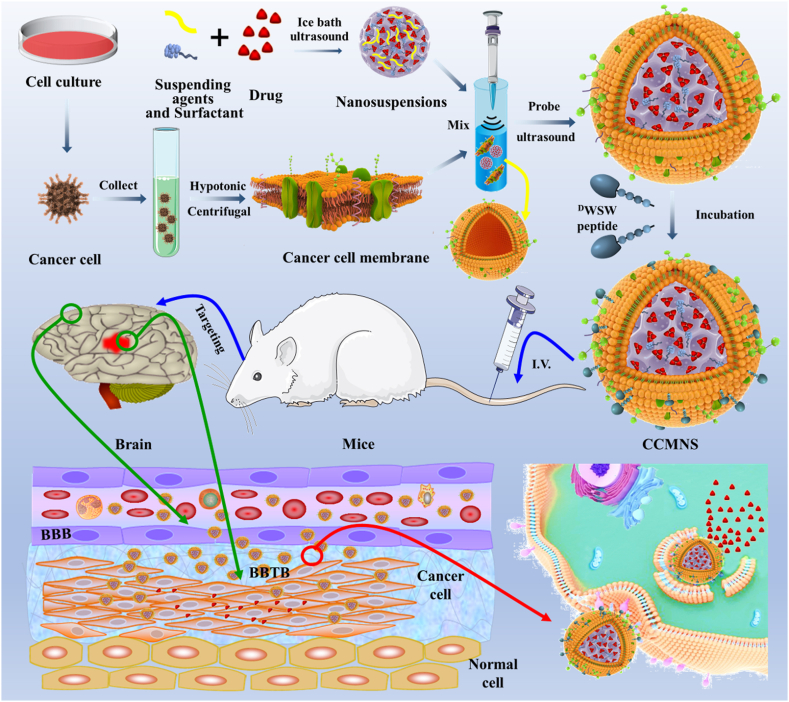
Graphic abstract of this study. Firstly, paclitaxel nanosuspensions were prepared by the ultrasonic-precipitation method. Secondly, the cancer cells were cultured and collected, and the cell membrane was separated from the plasma membrane of the source cells by hypotonic and differential centrifugation. Secondly, the cancer cells were cultured and collected, and the nuclei and membranes were separated by hypotonic and differential centrifugation. The obtained cancer cell membranes were combined with the nanosuspensions, and the mixture was fused by an ultrasonic probe to construct a biomimetic nanosuspension. The targeted ligand ^D^WSW peptide was modified on the surface of the cell membranes by lipid insertion. Mice with *in situ* glioma were treated by intravenous injection, and the nanosuspensions penetrated the blood-brain barrier and the drug was delivered to the tumor to treat it.

Glioma is a common malignant tumor. Due to the BBB and the blood-brain-tumor-barrier (BBTB), drugs cannot cross the physiological and pathological barriers, and thus, many therapeutic drugs cannot enter the brain [16]. Finding ways to enable a drug to penetrate the BBB into the brain parenchyma to reduce systemic toxicity and penetrate the BBTB to exert a therapeutic effect has become a challenge [[17], [18], [19]]. In this study, biomimetic nanosuspensions with receptor-mediated endocytosis were constructed (Fig. 1). The drug nanosuspension was coated with C6 glioma cell membranes and modified with an active targeting ligand for the delivery of anticancer drugs [20,21]. Specifically, using the technology of nanometerization of poorly soluble drugs, a paclitaxel-nanosuspension (PTX)NS was prepared by the ultrasonic-precipitation method. Then, C6 cancer cell membranes (CCMs) were fused to the (PTX)NS to obtain CCM-(PTX)NS. Finally, the ligand D-type WSW (^D^S^D^Y^D^P^D^G^D^W^D^S^D^W) peptide was modified on the surface of tumor cell membranes by the lipid insertion method. WSW (also called PhrCACET1) is a peptide originating from *Clostridium acetobutylicum*. It can effectively cross the BBB and showed measurable tumor-targeting [22,23]. ^D^WSW-CCM-(PTX)NS could effectively penetrate the BBB and increase drug accumulation in the tumor site after administration to glioma-bearing mice. This provides an effective platform for the targeted drug delivery of (PTX)NS to glioma.

## 2 Materials and methods

### 2.1 Materials

Polyvinylpyrrolidone K30 (PVP K30) and sodium deoxycholate (SDC) were provided by Fenglijingqiu Commerce and Trade Co., Ltd. (Beijing, China), and DSPE-PEG_2000_-NHS was purchased from Xi'an ruixi Biological Technology Co., Ltd (Shanxi, China). Anti-CD44, anti-CD47, and anti-CD31 antibodies were purchased from Abcam (Cambridge, UK). PTX was obtained from Chendu Dsiter Co., Ltd (Sichuan, China). All chemical reagents were of analytical grade and purchased from Macklin Biochemical Co., Ltd.

### 2.2 Cells and experimental animals

Rat C6 glioma cells, bEnd.3 cells (mice brain microvascular endothelial cells), and human umbilical vein endothelial cells (HUVEC) were supplied by the Cell Resource Centre of IBMS (Beijing, China) and cultured in Dulbecco's modified Eagle's medium (DMEM) containing 10% fetal bovine serum (FBS) (Gibco) and 100 IU of penicillin.

Female and male ICR mice weighing 18–22 g were purchased from SPF Biotechnology Co., Ltd. (permit number: SCXK (Jing) 2019-0010, Beijing, China). All procedures involving the care and handling of animals were carried out with the approval of the Animal Care and Use Ethics Committee of the Beijing Institute of Pharmacology and Toxicology (Beijing, China).

### 2.3 Preparation of nanosuspensions

(PTX)NS was prepared by the ultrasound precipitation method [24]. Briefly, 20 mg of PTX was dissolved in 1 mL of ethanol as the oil phase, and 20 mg of PVP K30 and 60 mg of SDC were dissolved in 20 mL water as the water phase. The water phase was placed into an ice bath ultrasound (840 W) and the oil phase was slowly dropped into the water phase and stirred while dropping. After dropping, ultrasonification was continued for 5 min, and the volatile organic solvent was magnetically stirred under low-temperature conditions to obtain (PTX)NS. The nanosuspension was stored at 4 °C for later use.

### 2.4 Synthesis of targeting ligands

Targeting ligands were synthesized by the active ester method. With N-hydroxythiosuccinimide (NHS) as a protectant, the ester bond in DSPE-PEG_2000_-NHS was reacted with the amino group in the ^D^WSW peptide under the catalysis of N,N′-dicyclohexylcarbodiimide (DCC) to form an amide bond. The targeted ligand was obtained by lyophilization after dialysis. Nuclear magnetic resonance (^1^H-NMR) and MALDI-TOF mass spectrometry (MALDI-TOF MS) were used to characterize the synthesis of the targeting ligands [25].

### 2.5 Isolation of C6 cancer cell membranes

C6 cells were cultured in DMEM medium containing 10% FBS (37 °C, 5% CO_2_) as previously described [[26], [27], [28]]. When the cell density reached 80%–90%, the cells were trypsinized and collected by centrifugation at 2000 RPM for 5 min and washed with phosphate-buffered saline (PBS, pH 7.4). The cells were isolated in ice-cold 25% PBS (pH 7.4) containing 0.5 mM EDTA and 30 mM Tris-HCl supplemented with protease inhibitor cocktail. After 30 min, the cells in solution were broken by ultrasonic waves for 2 min (300 W; sonication for 2 s, stopping for 5 s) and then centrifuged at 20000 RPM at 4 °C for 10 min to remove the nuclei and organelles, etc. The supernatant was centrifuged again at 100,000 RPM at 4 °C for 30 min [29]. CCMs were collected and stored at −80 °C for further use.

### 2.6 Preparation and modification of CCM-encapsulated nanosuspensions

The prepared cell membranes were resuspended with 5 mL of the nanosuspension and fused using an ultrasonic cell disruptor (300 W; sonication for 2 s, stopping for 5 s) in an ice bath for 5 min to obtain cancer cell membrane-encapsulated nanosuspensions (CCM-(PTX)NS). Then, targeted modification was carried out by lipid insertion. Briefly, 2 mg of DSPE-PEG_2000_-^D^WSW ligand was mixed with the biomimetic nanosuspensions and stirred in an ice bath for 8 h to obtain ^D^WSW-CCM-(PTX)NS. 1,1′-Dioctadecyl-3,3,3′,3′-tetramethylindocarbocyanine perchlorate (DiI) or 1,1′-Dioctadecyl-3,3,3′,3′-tetramethylindotricarbocyanine iodide (DiR)-labeled CCM-(PTX)NS and ^D^WSW-CCM-(PTX)NS was obtained by adding free DiI or DiR to the biomimetic nanosuspensions and stirring for 20 min in the dark. Free DiI and DiR were removed by dialysis for 12 h in the dark.

### 2.7 Characterization of nanosuspensions

The size and zeta potential of the fabricated (PTX)NS, CCM-(PTX)NS, and ^D^WSW-CCM-(PTX)NS were determined by dynamic light scattering (DLS, Litesizer™ 500, Anton-Paar, Austria). The nanosuspensions were stored at 4 °C for 7 days and stability was observed [30].

Drug powder was prepared by the freeze-drying method. Briefly, 20 mL of (PTX)NS was placed in a 50 mL beaker, pre-frozen at −80 °C for 4 h, and then freeze-dried for 24 h to obtain solid (PTX)NS. PTX, PVP K30, and SDC physical mixtures and solid (PTX)NS were analyzed by X-ray diffraction (XRD, D8 advance, Bruker, Germany) at 2°/min. Scanning angles of 5–90° were used to observe changes in the drug crystals. The physical state and the crystal form of the drug were further confirmed by differential scanning calorimetry (DSC, DSC204 HP, Netzsch, Germany). The heating program was 10 °C/min and the temperature range was 35–235 °C. Then, Fourier transform infrared (FTIR, Spectrum Two, PerkinElmer, USA) spectroscopy was used to analyze and identify the drug to characterize whether the drug molecule had changed (wavelengths from 4000 to 450 cm^−1^).

For microscopic morphology observation, a small amount of PTX was taken and spread on conductive tape. The sample was sprayed with gold using ion sputtering equipment and observed under scanning electron microscopy [31] (SEM, S4800, Hitachi, Japan). At the same time, a small amount of (PTX)NS, CCM-(PTX)NS, and ^D^WSW-CCM-(PTX)NS were dripped onto copper mesh (300 mesh). The solvent was evaporated and stained with 1% phosphomolybdic acid for 1 min [32,33]. The morphology was observed under transmission electron microscopy (TEM, HITACHI, H-7650, Japan).

### 2.8 Protein determination

The membrane protein of the C6 cell membranes was detected by sodium dodecyl sulfate-polyacrylamide gel electrophoresis (SDS-PAGE) to check whether the membrane protein was lost in the preparation process [14,34]. Five milliliters of (PTX)NS, CCM, CCM-(PTX)NS, and ^D^WSW-CCM-(PTX)NS were collected by centrifugation at 4 °C at 15,000 RPM for 10 min. After removing the supernatant, 0.5 mL of protein lysate was added, and the protein concentration was determined by the bicinchoninic acid (BCA) method [35]. Then, the sample was analyzed.

Specific membrane proteins are signs of the functional integrity of the cell membrane. CD47 is expressed on the surface of cancer cells and is generally considered to be a receptor protecting cancer cells from the host immune system [36,37]. CD44 is a glycoprotein on the cell surface, which participates in cell-cell interaction, cell adhesion, and cell migration, and its expression increases in malignant tumors [36,37]. The expression of the specific membrane markers was evaluated by Western blots (WB). The secondary structure of proteins plays an important role in maintaining the physiological activity of proteins. Circular dichroism (CD, DSM 1000, OLIS, USA) was used to determine changes in the secondary structure of the proteins in different preparations to further clarify the activity of the membrane proteins.

### 2.9 *In vitro* release

The *in vitro* release of PTX was performed in PBS at pH 7.4 and pH 6.8 (containing 0.5% Tween-80). One milliliter of PTX, (PTX)NS, CCM-(PTX)NS, and ^D^WSW-CCM-(PTX)NS were placed into a 50 mL triangular bottle and shaken at 37 °C at 100 RPM. Two-milliliter samples were taken at 0, 5, 10, 15, 20, 25, 30, 40, 50, and 60 min, and an equal amount of preheated release medium was added immediately. The solution was filtered through 0.2 μm microporous film and the drug content was determined by high-performance liquid chromatography (HPLC, 1200, Agilent, USA). The cumulative percentage of drug release was calculated, and the *in vitro* release curve was drawn.

### 2.10 Cell uptake

To investigate the targeting ability of biomimetic nanosuspensions, three cell lines, bEnd.3 cells, HUVEC cells, and C6 cells were selected to evaluate DiI-labeled particle endocytosis efficiency. Cells (10,000) were placed in a culture dish (20 mm) and observed under an inverted microscope until the cells adhered to the dish. Biomimetic nanosuspensions (200 μL) were incubated for 15 min with the cells in 5% CO_2_ at 37 °C [38]. After that, the cells were fixed with 4% paraformaldehyde solution or collected with pancreatin, and the nuclei were stained with Hoechst 33258. Finally, the cells were sealed with 50% glycerin [13]. The uptake was measured by confocal laser scanning microscopy (CLSM, LSM 880, Zeiss, Germany) and quantitative analysis was performed by flow cytometry (FCM, FACSAria III, BD, USA).

### 2.11 *In vitro* cytotoxicity

The Cell Counting Kit-8 (CCK-8) was used to determine the inhibitory effect of nanosuspensions on tumor cell proliferation. Logarithmic phase C6 cells were collected and 5000 cells were seeded and differentiated in 96-well plates at 37 °C in a 5% CO_2_ atmosphere. Different drug solutions containing 2, 5, 20, 50, 100, or 200 g/mL of PTX were added to each group. After 48 h of culture, 20 μL of CCK-8 solution was added to each well and incubated for 2 h. The absorbance of each well was measured at 450 nm using a plate reader (Tecan Spark, Austria).

### 2.12 Homotypic targeting

Four model cancer cell lines, mouse breast cancer cells (4T1), mouse melanoma cells (B16), human hepatoma cells (HepG2), and C6 cells were selected to study *in vitro* cellular uptake. Each type of tumor cell was incubated in culture dishes (20 mm) for 12 h. The cells were incubated with DiI-CCM-(PTX)NS for 0.5 h. The cell samples were washed 3 times with PBS for 5 min and fixed with 4% polyformaldehyde for 20 min. Then, the cells were stained with Hoechst 33258 for 20 min [13,32,33,39]. Finally, the cells were observed by CLSM.

### 2.13 Transport across the BBB and BBTB

The *in vitro* BBB model was established according to the literature [35]. To summarize, bEnd.3 cells were seeded onto the upper chamber of cell culture inserts (4 μm)at a density of 1.0 × 10^5^ cells per well. After culturing until the cells were 100% confluent, the transendothelial electrical resistance (TEER) of the cell membrane was recorded. When the TEER was over 300 Ω cm^2^, the culture dish was inoculated with 1.0 × 10^5^ C6 cells, and the cell culture chamber (upper chamber) was inserted into the culture dish (bottom chamber) after the cells adhered [40,41]. Free DiI, DiI-CCM-(PTX)NS, and DiI-^D^WSW-CCM-(PTX)NS were added to the upper chamber and cultured for 4 h. The solutions collected from the bottom chamber were detected by a plate reader. The cell uptake was observed by CLSM. Similarly, HUVEC cells and C6 cells were used to establish a BBTB model to investigate the penetration ability of the preparations.

The inhibitory effect of PTX on glioma cells in the BBB and BBTB models *in vitro* was determined using Transwell chambers (Corning, NY, USA). Simply, 1 × 10^5^ bEnd.3 cells were seeded on the apical Transwell chamber and 2000C6 cells were inoculated on the basal chamber when the cells were confluent and the TEER was over 300 Ω cm^2^. Free PTX, CCM-(PTX)NS, and ^D^WSW-CCM-(PTX)NS (at PTX concentrations of 50 μg/mL) were added to the apical chamber, cultured for 48 h, and the CCK-8 method was used to determine the inhibition of the C6 cells.

### 2.14 Targeting ability in the intracranial glioma-bearing mice model

According to a previous report [42,43], 1 × 10^7^ C6 cells were intracerebrally injected into the brain of ICR mice. The mice were divided into four groups and injected with 100 μL of normal saline, DiR, DiR-CCM-(PTX)NS, or DiR-^D^WSW-CCM-(PTX)NS [44]. At 2, 4, 8, 12, 24, and 36 h after administration, the mice were anesthetized with 3% isoflurane and analyzed (748/780 nm) using an IVIS *in vivo* system (IVIS® Spectrum, PerkinElmer, USA). Living Image® software (Caliper, Alameda, CA) was used to quantify the bioluminescence and fluorescence signals. The major organ tissues (heart, liver, spleen, lungs, and kidneys) were harvested, imaged, and analyzed *in vitro* using the same method [[45], [46], [47]].

For the tumor distribution analysis, tumor-bearing mice were injected with normal saline, DiI, DiI-CCM-(PTX)NS, or DiI-^D^WSW-CCM-(PTX)NS via the tail vein. The mice were euthanized 4 h after the administration, the brains were removed, placed in 4% paraformaldehyde, and fixed for 24 h in the dark. Then, paraffin sections were cut. The cell nucleus was stained with 4′,6-diamidino-2-phenylindole (DAPI) (358/461 nm), and the distribution of the biomimetic nanosuspensions in the tissue was measured with a fluorescence microscope [48,49].

### 2.15 Targeting ability of ligands

Based on the specific binding between ligands and receptors, the glioma-bearing mice were divided into two groups. In the first group, free ^D^WSW peptide (150 μL, 1 mg/mL) was injected 1 h before the experiment, and then 150 μL of DiR-^D^WSW-CCM-(PTX)NS was injected into the two groups at the same time for *in vivo* imaging. The blocking of the receptors on the BBB by the ^D^WSW peptide was determined [[50], [51], [52]].

### 2.16 *In vivo* anti-glioma activity

The glioma-bearing mice were randomly divided into five groups (n = 8). Normal saline, PTX, (PTX)NS, CCM-(PTX)NS, or ^D^WSW-CCM-(PTX)NS was injected through the tail vein every two days with a PTX dose of 20 mg/kg. The survival time was recorded and Kaplan-Meier estimation was used to analyze the survival rate [53]. At the same time, an experimental group was set up for 10 days of administration. After administration, brain glioma was evaluated by magnetic resonance imaging (MRI) (PharmaScan 70T/16, Bruke, US); the mice were euthanized; and the brains were removed [54]. The brains were fixed in 4% paraformaldehyde for two days. The whole brain was embedded in a wax block after dehydration and sectioned for hematoxylin and eosin (HE) staining, the TUNEL assay, and CD31 immunohistochemical detection. The damaging effects of the different drug groups on tumor tissue were determined [55].

### 2.17 *In vivo* toxicity evaluation

To detect the potential *in vivo* toxicity of the nanosuspensions, normal mice (male) were randomly divided into five groups. Normal saline, PTX, (PTX)NS, CCM-(PTX)NS, or ^D^WSW-CCM-(PTX)NS was injected through the tail vein every two days for 15 consecutive days with a PTX dose of 40 mg/kg. All mice were euthanized on the second day after the last administration, and blood cell and serum biochemical indicators were analyzed. The heart, liver, spleen, lung, kidney, and brain were removed for histopathological examination (HE staining) [12,56].

### 2.18 Statistical analysis

Quantitative data are expressed as mean ± standard deviation (SD) unless otherwise indicated. One-way analysis of variance (ANOVA) was used to determine significant differences between different groups, and p < 0.05 was considered to indicate statistical significance.

## 3 Results and discussion

### 3.1 Characterization of (PTX)NS

**Fig. 2:**
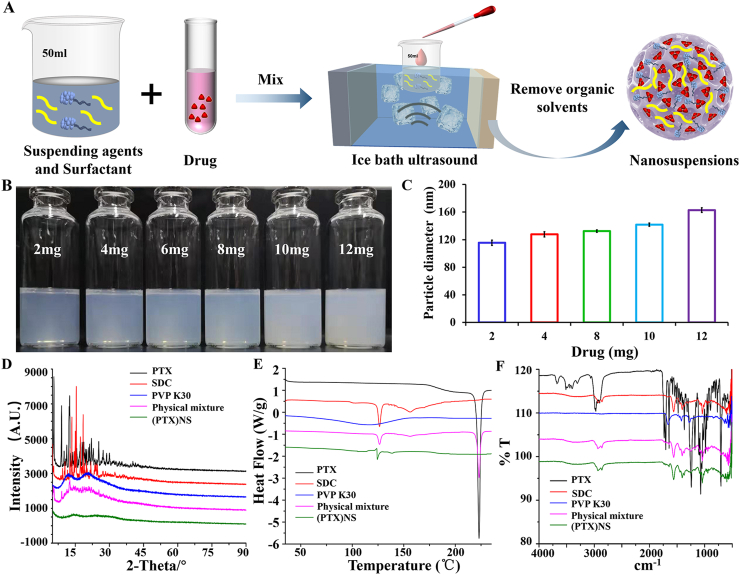
Preparation and characterization of paclitaxel nanosuspension. (A) The suspending agent and the surfactant were dissolved in the water phase, the drug was dissolved in the oil phase, and the nanosuspension was prepared by the ultrasonic-precipitation method. (B) Nanosuspensions with different drug amounts where higher doses showed lower light transmission. (C) The particle size of the nanosuspensions with different drug amounts, showing the higher the dose, the larger the particle size. (D) X-ray diffraction patterns, (E) DSC scanning images, and (F) FTIR spectra of different components in the nanosuspensions.

The preparation process of (PTX)NS is illustrated in Fig. 2A. With an increase in drug-loading, the particle size of the nanosuspensions gradually increased (Fig. 2B and C). It can be seen from the XRD (Fig. 2D) that PTX and SDC showed strong crystal diffraction peaks, which were not present in PVP K30. This indicated that PTX and SDC were crystalline structures, whereas PVP K30 was not. The crystal diffraction peak also appeared in the physical mixture. However, the crystal diffraction peaks completely disappeared in the nanosuspensions, indicating that PTX and SDC in (PTX)NS changed to amorphous forms. The same sample was analyzed by DSC (Fig. 2E). PTX demonstrated a sharp endothermic peak at 223.09 °C correspondings to its melting point and the peak in (PTX)NS disappeared, indicating that the crystalline state of PTX was probably transformed into an amorphous state, consistent with the XRD results. The determination of the drug structure in the nanosuspensions by FTIR is shown in Fig. 2F. The distinctive peaks of PTX were maintained in (PTX)NS, confirming that the original composition of the drug was retained. The characterization results proved the successful preparation of (PTX)NS.

### 3.2 Characterization of biomimetic nanosuspensions

**Fig. 3:**
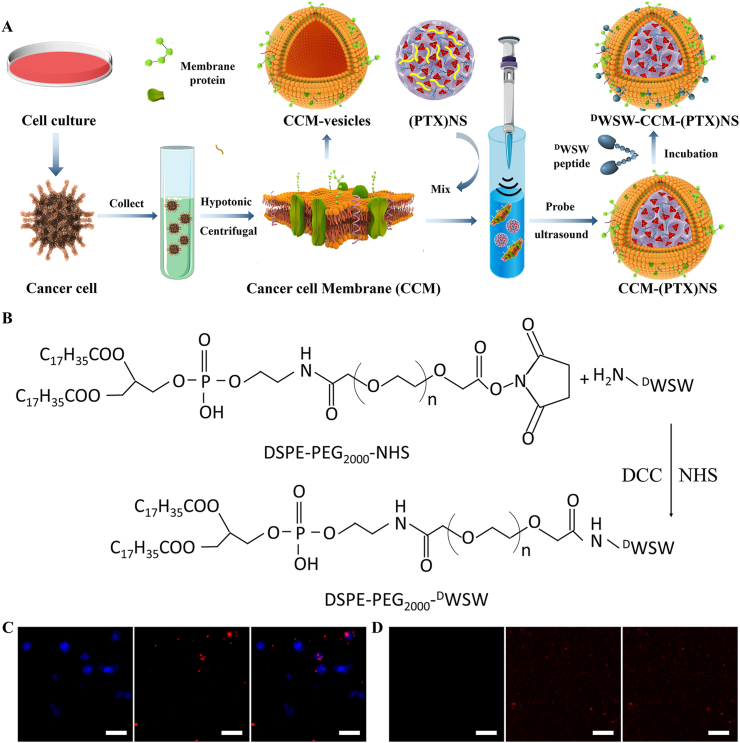
(A) Preparation of PTX biomimetic nanosuspension. Cells were collected after culturing, and hypotonic treatment and centrifuging were used to obtain cell membranes. The membranes were combined with (PTX)NS using ultrasonic fusion to obtain biomimetic nanosuspensions and active targeting modification was carried out by the lipid insertion method. (B) Synthesis of targeting ligand DSPE-PEG_2000_-^D^WSW using the active ester method. In the presence of DCC and NHS, the –NH_2_ in the polypeptide reacted with the active ester in DSPE-PEG_2000_-NHS. (C) Cell nuclei obtained by low-speed centrifugation in the cell membrane preparation. (D) Cell membranes after purification. Hoechst 33258 (blue), DiI (red), (60x magnification).

As shown in Fig. 3A, the probe ultrasound method to fuse cancer cell membranes with the drug nanosuspension was simple and reproducible. The synthetic route of DSPE-PEG_2000_-^D^WSW is shown in Fig. 3B, and the fluffy powder-targeting ligand was obtained by freeze-drying. MALDI-TOF MS confirmed the successful synthesis of DSPE-PEG_2000_-^D^WSW. A molecular ion peak with an *m/z* of 3816.41 (Appendix A, Appendix A) was seen, which was equivalent to the molecular weight of DSPE-PEG_2000_-^D^WSW. The distance between each peak was about 44 D, which was the polymer repeating unit (-CH_2_-CH_2_-O-)_n_. ^1^H-NMR (Appendix A, Appendix A) also showed the successful synthesis of the target molecule, the characteristic peak of DSPE-PEG_2000_-NHS disappeared, and the characteristic peak of ^D^WSW appeared (δ = 3.51). CLSM was used to observe the separation of the nucleus and membrane. Fig. 3C shows that the nuclei had been centrifuged out and some cell membranes had also been collected. In Fig. 3D, it was obvious that the nuclei had been removed and the cell membranes were purified.

The particle size and zeta potential measurement results are shown in Appendix A, Appendix A. The particle size of the different nanosuspensions was uniform and the polydispersity index (PDI) was small. The particle size of (PTX)NS was about 144 nm, and that of the cancer cell membrane-encapsulated nanoparticles was 169 nm. The particle size of the biomimetic nanosuspensions increased to about the thickness of a cell membrane. As the zeta potential of biomimetic nanosuspensions increases, they become more stable in liquid. Furthermore, the targeted modification did not change the particle size or potential of the biomimetic nanosuspensions.

**Fig. 4:**
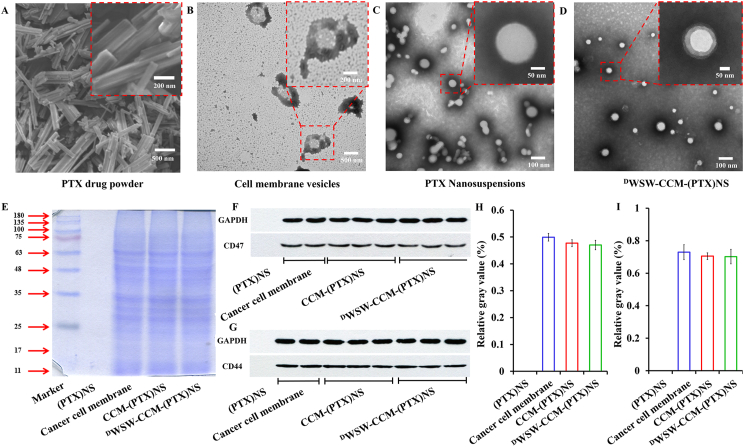
Characterization of biomimetic nanosuspensions. (A) SEM of paclitaxel powder. The drug had a crystalline structure. Cell membrane vesicles (B), (PTX)NS(C) and ^D^WSW-CCM-(PTX)NS(D) transmission electron microscopy images. The drug nanosuspension was approximately spherical and had a core-shell structure after being wrapped by the cancer cell membranes. (E) The membrane protein of the biomimetic nanosuspensions was determined by SDS-PAGE. WB measurement and relative gray value of membrane-specific proteins CD47 (F and H) and CD44 (G and I).

The SEM results of the drug powder are shown in Fig. 4A. The drug had a clear crystal structure, consistent with the XRD and DSC results. As can be seen from the TEM results in Fig. 4B, the cell membrane vesicles have no core and most of the drug had spherical structures of uniform size after being prepared into the nanosuspensions, and the particle size was close to the measured value (Fig. 4C and D). The drug nanoparticles in the nanosuspensions wrapped by the cancer cell membrane were spherical. A layer of membrane structure was seen on the surface of the nanoparticles, which had a core-shell structure [5,57].

The membrane proteins of cancer cells were analyzed by the SDS-PAGE method. As shown in Fig. 4E, no protein bands were found in (PTX)NS. However, all the preparations containing cancer cell membranes showed protein bands at different molecular weight maker protein positions, and their protein profile closely matched that of the purified membranes. The protein contents of (PTX)NS, CCM-(PTX)NS, and ^D^WSW-CCM-(PTX)NS were quantified using the BCA kit (Appendix A, Appendix A). The cell membrane transmembrane CD47 target protein can bind to SIRPα on the surface of macrophages, which can be recognized by the immune system as its own substance and reduce phagocytosis. As a transmembrane glycoprotein, CD44 is widely involved in the heterogeneous adhesion of tumor cells and is also a marker protein of tumor cells. As shown in Fig. 4F and G, there was no expression of CD47 and CD44 in (PTX)NS. However, the nanosuspension wrapped by cancer cell membranes all showed the expression of target protein, and the bands were consistent. The gray value analysis showed that the membrane protein was basically not lost (Fig. 4H and I). Also, the secondary structure of the protein was determined by CD (Fig. 2B), and the 3D structure of the protein was not destroyed. These results indicated that these biomimetic nanosuspensions retained the characteristics of the cancer cell membranes.

The release results of different preparations are shown in Appendix A, Appendix A (pH 7.4) and Appendix A, Appendix A (pH 6.8). The specific surface area of the drug became larger after nanometerization, which improved the release rate. In the two solutions, the release rate of nanosuspensions was more than 60% in 20 min. Compared to (PTX)NS, CCM-(PTX)NS, and DWSW-CCM-(PTX)NS, the drug release rate of the biomimetic nanosuspensions was not affected and the targeted modification was not affected either. Furthermore, Appendix A, Appendix A shows that at 4 °C, (PTX)NS, CCM-(PTX)NS, ^D^WSW-CCM-(PTX)NS remained stable for seven days. The effect of PTX, (PTX)NS, CCM-(PTX)NS, and ^D^WSW-CCM-(PTX)NS to inhibit the proliferation of C6 cells was investigated using the CCK-8 assay (Appendix A, Appendix A). The results showed that each group had a significant dependence on concentration. With the increase in drug concentration, the inhibition of cell proliferation was significantly enhanced. The IC50 values of PTX, (PTX)NS, CCM-(PTX)NS, DWSW-CCM-(PTX)NS were 37.50, 34.36, 9.51, and 7.76 μg/mL, respectively, and ^D^WSW-CCM-(PTX)NS had the strongest effect.

### 3.3 Cellular uptake of biomimetic nanosuspensions

**Fig. 5:**
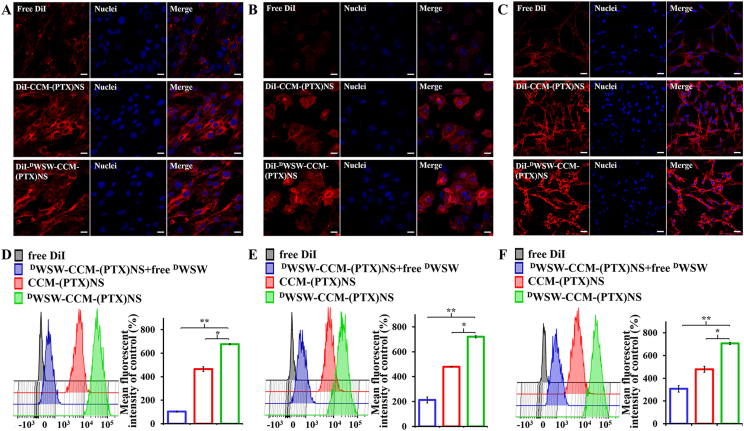
Cell uptake measured by CLSM and FCM. CLSM images and the corresponding flow cytometry analysis of bEnd.3 (A and D), HUVEC (B and E), and C6 (C and F) cells after incubation with DiI, DiI-CCM-(PTX)NS, or DiI-^D^WSW-CCM-(PTX)NS. The preparations showed different targeting capabilities. (20x magnification; red: DiI, blue: nuclei; *p < 0.05, **p < 0.01).

As shown in Fig. 5A and D, CCM-(PTX)NS and ^D^WSW-CCM-(PTX)NS had high intracellular fluorescence intensity and could be effectively taken up by bEnd.3 cells, indicating that the C6 cancer cell membranes had the ability to penetrate the BBB. Meanwhile, the uptake of biomimetic nanosuspensions was the highest after targeted modification, while free ^D^WSW peptide could significantly inhibit the uptake of ^D^WSW-CCM-(PTX)NS, which indicated that ^D^WSW peptide had good brain targeting and could transport drugs to the brain.

To verify the targeting ability of the nanosuspensions to tumor tissues, the cellular uptake of HUVECs, which is similar to tumor neovascular endothelial cells, was measured. As shown in Fig. 5B and E, camouflaging (PTX)NS with the C6 cancer cell membranes significantly increased the cellular uptake into HUVEC cells. Meanwhile, free ^D^WSW peptide could also significantly inhibit the uptake of ^D^WSW-CCM -(PTX)NS, indicating that the ^D^WSW peptide could transport drugs to tumors by targeting tumor angiogenesis.

Finally, the targeting and uptake ability of biomimetic nanosuspensions in C6 cells were studied (Fig. 5C and F). CCM-(PTX)NS was taken up more by C6 cells, which demonstrated homologous targeting between the tumor cells. ^D^WSW-CCM-(PTX)NS showed the highest level of cellular uptake. This proved that while ^D^WSW-CCM-(PTX)NS had homologous targeting, it also showed active targeting, and dual-targeting was more conducive to transporting drugs to tumor cells.

### 3.4 Homologous targeting

To verify the targeting ability of CCM-(PTX)NS to homotypic cancer cells, the intracellular uptake of CCM-(PTX)NS was evaluated in B16 (Appendix A, Appendix A), HepG2 (Appendix A, Appendix A), 4T1 (Appendix A, Appendix A), and C6 (Appendix A, Appendix A) cancer cells. In B16, HepG2, and 4T1 cells, the red fluorescence intensity was weaker than that of the C6 cells, which further demonstrated that C6 cell membranes could significantly facilitate uptake of the cancer cell membrane source, suggesting that the remaining proteins on the cell membranes coated on the (PTX)NS retained the primary recognition proteins.

### 3.5 *In vitro* BBB and BBTB models

The BBB *in vitro* model was successfully established with bEnd.3/C6 cells and the resistance value was 313.2 Ω·cm^2^. It can be seen from Appendix A, Appendix A that the penetration ability of ^D^WSW-CCM-(PTX)NS was the strongest, and the fluorescence intensity of the bottom chamber solution was the highest (Appendix A, Appendix A). There was a significant difference compared to CCM-(PTX)NS, which proved the importance of a targeting peptide as a ligand for crossing the BBB. The HUVEC/C6 cell co-culture model was used as the BBTB, and the resistance value was 321.5 Ω cm^2^. ^D^WSW-CCM-(PTX)NS showed the strongest targeting capability, consistent with the BBB results (Appendix A, Appendix A). The apoptosis rate of C6 cells in the BBB and BBTB was determined using the CCK-8 kit. As shown in Appendix A, Appendix A, the cell survival rates of ^D^WSW-CCM-(PTX)NS, CCM-(PTX)NS, (PTX)NS, and PTX in the BBB were 51.13, 71.67, 84.21, and 93.39% respectively, and those in the BBTB were 57.373, 74.57, 84.93, and 92.50% respectively. The biomimetic nanosuspensions modified by the targeting peptide demonstrated the highest inhibition of cancer cell proliferation.

### 3.6 *In vivo* imaging and biodistribution

**Fig. 6:**
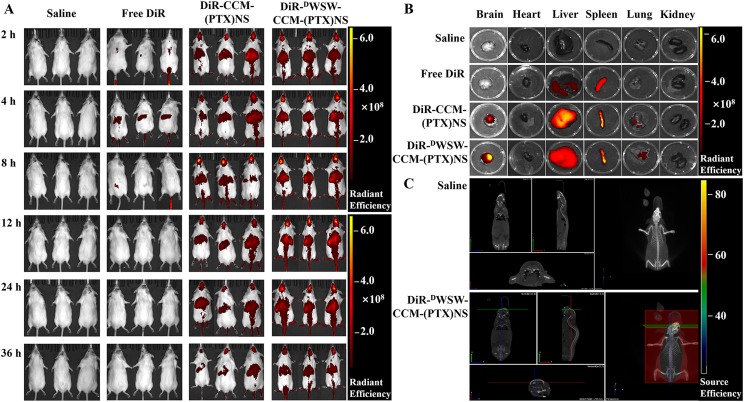
*In vivo* targeting evaluation. (A) *In vivo* real-time imaging of saline, DiR, CCM-(PTX)NS, and ^D^WSW-CCM-(PTX)NS in glioma-bearing mice (n = 3). (B) Biodistribution of different DiR-encapsulated nanosuspensions in different organs. (C) 3D brain CT tumor localization scan. This indicated that the biomimetic nanosuspensions could effectively transport drugs to brain tissue.

After the carrier was labeled with DiR, the distribution of the biomimetic nanosuspensions in the body could be visually observed using the *in vivo* imaging system. As shown in Fig. 6A, the fluorescence signal of the tumor-bearing mice in the normal saline group was weak, which did not affect the measurements of the experimental group at the same fluorescence level. CCM-(PTX)NS could penetrate the BBB and be transported into brain tissue, which showed homologous targeting ability. With the extension of time, the brain volume increased first and then decreased, and the highest concentration was detected at 12 h. ^D^WSW-CCM-(PTX)NS showed the strongest brain-targeting ability, with the highest concentration at 12 h. The brain fluorescence intensity analysis showed that ^D^WSW-CCM-(PTX)NS had an obvious targeting advantage (Appendix A, Appendix A).

The imaging analysis of the isolated tissue showed that the fluorescence intensity of the brain tissue was in the order of ^D^WSW-CCM-(PTX)NS > CCM-(PTX)NS (Fig. 6B and Appendix A, Appendix A). The ^D^WSW peptide-modified biomimetic nanosuspension had the highest concentration and the strongest targeting ability in brain tissue, which confirmed the important role of peptide modification. Among the other tissues, the fluorescence intensity was strongest in the liver and spleen. The liver, as the largest elimination organ, accumulated the biomimetic nanosuspensions. The spleen is the largest immune organ. Immune cells captured the nanosuspensions in peripheral tissues and transported them to the spleen. The accumulation of ^D^WSW-CCM-(PTX)NS in the tumor site was confirmed by 3D CT scanning fluorescence imaging (Fig. 6C), which again verified the brain-targeting property of the ^D^WSW peptide.

The free ^D^WSW peptide was used to block the related receptors of the mice BBB to verify the brain targeting of the ligand. Appendix A, Appendix A clearly show that after the ^D^WSW peptide blocked the BBB receptor, the transport of ^D^WSW-CCM-(PTX)NS was significantly reduced. However, some drugs were still transported to the brain tissue under the homologous targeting mechanism.

**Fig. 7:**
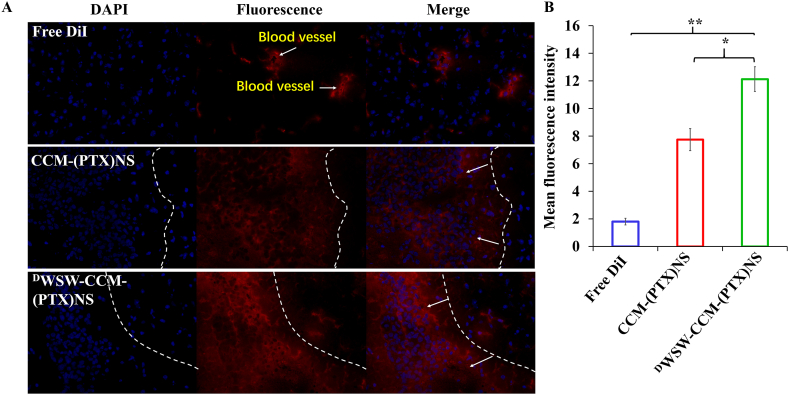
(A) Distribution of brain tissue *in vitro*. ^D^WSW-CCM-(PTX)NS had the highest fluorescence intensity and best targeting in tumor tissues. ImageJ was used for quantification (B). (DAPI: blue, DiI: red; 20x magnification; *p < 0.05, **p < 0.01).

The brain tissue of mice injected with DiI-labeled biomimetic nanosuspensions was sectioned. As can be seen from Fig. 7A, free DiI did not enter the tumor tissues, but CCM-(PTX)NS and ^D^WSW-CCM-(PTX)NS were distributed in the tumor tissues, confirming their homologous targeting effect. The ligand-modified biomimetic nanosuspension had a better targeting effect. ImageJ quantitation also indicated that ^D^WSW-CCM-(PTX)NS had the greatest distribution in tumor tissues (Fig. 7B).

### 3.7 *In vivo* anti-glioma evaluation

**Fig. 8:**
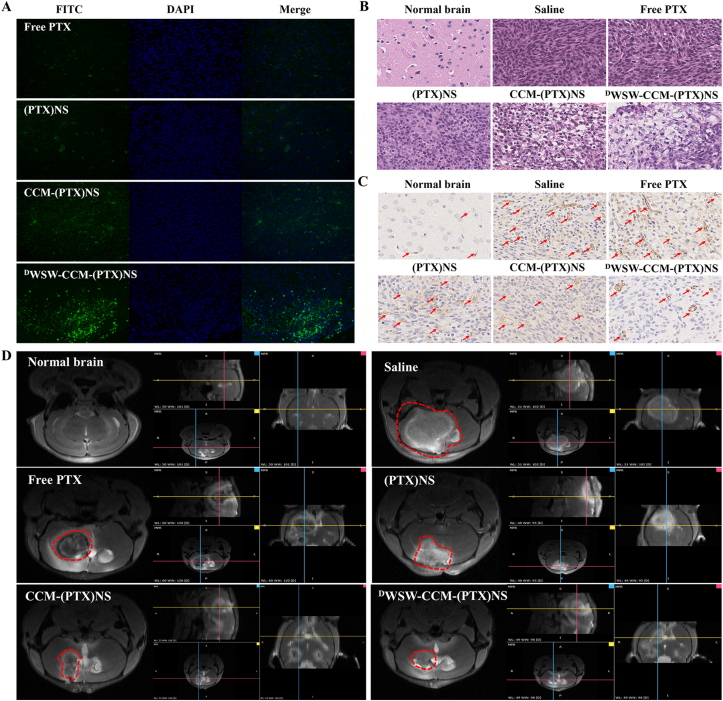
*In vivo* anti-tumor effects in C6 glioma-bearing mice. (A) TUNEL staining of the tumors. (B) H&E staining of the tumor tissues. (C) Immunohistochemical staining of CD31 in the tumor tissues. (D) MRI of normal and glioma brains after treatment. The ^D^WSW-CCM-(PTX)NS group showed the strongest anti-tumor effect. (DAPI: blue, FITC: green, CD31: brown; 40x magnification).

The therapeutic effect of biomimetic nanosuspensions *in vivo* was evaluated in the glioma-bearing mice model. After treatment with the various tested formulations, ^D^WSW-CCM-(PTX)NS showed the best anti-tumor ability, which was significantly better than that of the CCM-(PTX)NS, (PTX)NS, and PTX groups. The Kaplan-Meier survival curves (Appendix A, Appendix A) showed that ^D^WSW-CCM-(PTX)NS significantly prolonged the lifespan of the mice. TUNEL staining and fluorescence intensity measurements showed (Fig. 8A and Appendix A, Appendix A) that, compared to the CCM-(PTX)NS, (PTX)NS, and PTX groups, tumor tissue cells treated with ^D^WSW-CCM-(PTX)NS significantly induced apoptosis (green). This confirmed that biomimetic nanosuspensions could inhibit tumor growth by inducing apoptosis.

Whole-brain H&E staining analysis showed that compared to the saline, PTX, (PTX)NS, and CCM-(PTX)NS groups, tumor cells treated with ^D^WSW-CCM-(PTX)NS had the least growth and the best tumor inhibition (Fig. 8B). Immunohistochemical staining of brain tissue was performed to investigate the expression of CD31 receptors in new blood vessels related to tumor cell proliferation. Compared to the saline, PTX, (PTX)NS, and CCM-(PTX)NS groups, the expression of CD31 in the ^D^WSW-CCM-(PTX)NS administration group was significantly decreased (Fig. 8C). Finally, the anti-glioma effect was evaluated by MRI and meglumine gadopentetate was used as a tumor imaging enhancer. Mice administered ^D^WSW-CCM-(PTX)NS had the smallest tumors. CCM-(PTX)NS also showed good anti-tumor effects under homologous targeting. The MRI 3D images showed the contours of the different tumor sections (Fig. 8D).

### 3.8 *In vivo* safety of biomimetic nanosuspensions

**Fig. 9:**
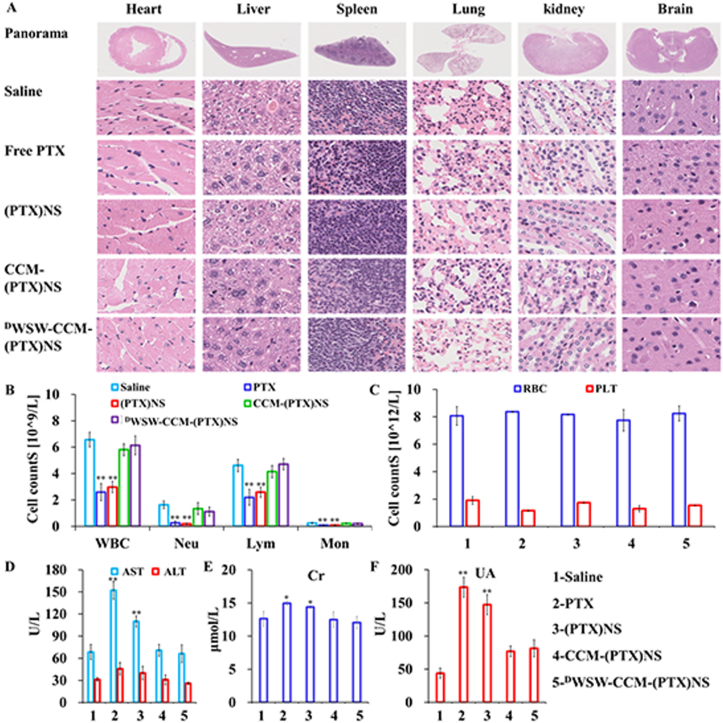
Preliminary safety evaluation. (A) Histological examination of the major organs derived from the mice after treatment. No major pathological changes were observed. Serum biochemical indicators of the mice after administration. (B) Immune system cells: WBC, Neu, Lym, and Mon counts. (C) Blood cells: RBC and PLT counts. (D) Liver and kidney function markers: AST, ALT, Cr, and UA. The data points represent the mean ± SD (n = 3). *p < 0.05, **p < 0.01; 40x magnification.

The biosafety of the biomimetic nanosuspensions was evaluated by histopathological and hematological analysis. Fig. 9A shows that after the administration of PTX, (PTX) ns, CCM-(PTX)NS, and ^D^WSW-CCM-(PTX)NS, there was no obvious pathological damage in the heart, liver, spleen, lung, kidney, and brain of the mice. The numbers of white blood cells (WBC), neutrophils (Neu), lymphocytes (Lym), monocytes (Mon), platelets (PLT), and red blood cells (RBC) in blood were determined (Fig. 9B and C). The indexes of each group of mice treated with CCM-(PTX)NS and ^D^WSW-CCM-(PTX)NS were relatively stable. Mice treated with PTX and (PTX)NS had increased WBC, Neu, Lym, and Mon counts in the blood during the treatment period. The blood levels of aspartate aminotransferase (AST), alanine aminotransferase (ALT), creatinine (Cr), and uric acid (Ua) in the PTX and (PTX)NS group remarkably increased (Fig. 9D–F). PTX and (PTX)NS treatment caused obvious damage to liver and kidney function, whereas the blood levels in mice treated with CCM-(PTX)NS and DWSW-CCM-(PTX)NS were relatively stable. This proved that nanosuspensions coated with cell membranes could effectively inhibit the leakage of drugs in the blood. CCM-(PTX)NS and ^D^WSW-CCM-(PTX)NS had good biocompatibility and their integrity was maintained in the blood circulation, which laid the foundation for drug delivery to tumors and effective anti-tumor effects.

## 4 Conclusions

In this study, paclitaxel nanosuspensions were successfully prepared by the ultrasonic-precipitation method. The preparation method was simple, reliable, and easy to repeat, which avoided the instability of nanosuspensions caused by high temperature produced by high-pressure homogenization and media milling methods. Compared to carrier nanopreparation, the drug-loading was significantly increased. Cancer cell membranes were successfully isolated and used to prepare biomimetic nanosuspensions, which were also modified with a targeted ligand. ^D^WSW-CCM-(PTX)NS showed good *in vivo* and *in vitro* targeting, significantly increased the growth inhibition of tumor cells, and prolonged the survival time of glioma-bearing mice.

Insoluble drugs have low solubility, which makes their adsorption difficult, resulting in low bioavailability. Nanosuspension technology is currently an effective means to solve this problem. It can improve bioavailability by changing the saturated solubility and dissolution rate of drugs. The technology can be used not only for water-insoluble drugs but also for drugs that are insoluble in both water and oil. The insoluble anti-tumor drug paclitaxel was nanocrystalized in this study to improve its concentration in an aqueous solution, which laid a foundation for its efficacy.

Cell membrane-encapsulated nanoparticle technology provides a new modification and camouflage strategy for the development of high-targeting and low-immunogenicity nanomaterials. It is hoped that the problem of the insufficient modification breadth of traditional nanoparticles can be solved and open up a new nanotherapy method for many diseases. The biomimetic nanosuspensions CCM-(PTX)NS designed in this study were composed of a glioma cancer cell membrane shell and an anti-cancer drug PTX core. With the help of the homologous targeting mechanism, a good molecular basis was provided for the targeting of biomimetic nanosuspensions.

Although cancer cell membranes have a certain targeting ability, their targeting ability is still relatively weak. The peptide-mediated tumor-targeting nano-drug delivery system has been widely studied due to its good targeting ability. Many peptides have shown good ability to cross the BBB and are widely used for brain drug delivery. As an effective ligand, the ^D^WSW peptide solved the accumulation of nanoformulations in gliomas. As revealed by the results of this study, ^D^WSW-CCM-(PTX)NS could penetrate the BBB, distribute to tumor tissue, and inhibit tumor cells in tumor-bearing mice.

In general, the superiority of ^D^WSW-CCM-(PTX)NS designed in this paper was verified at the cell and animal levels and showed a good therapeutic effect on glioma. This indicated that cancer cell membrane coating and ^D^WSW modification were effective in increasing the anti-glioma effect of paclitaxel nanosuspensions. This provides an effective strategy for the current targeted therapy of glioma and other tumors.

## CRediT authorship contribution statement

**Yueyue Fan:** Methodology, Validation, Investigation, Data curation. **Yuexin Cui:** Formal analysis, Writing – original draft, Writing – review & editing. **Wenyan Hao:** Conceptualization, Methodology, Software, Validation, Formal analysis. **Mengyu Chen:** Formal analysis, Data curation, Formal analysis, Investigation. **Qianqian Liu:** Investigation, Data curation. **Yuli Wang:** Data curation. **Meiyan Yang:** Data curation. **Zhiping Li:** Data curation, Meng Liang, Data curation. **Wei Gong:** Data curation. **Shiyong Song:** Conceptualization, Writing – original draft, Writing – review & editing, Supervision, Project administration. **Yang Yang:** Conceptualization, Writing – original draft, Writing – review & editing, Supervision, Project administration, Funding acquisition. **Chunsheng Gao:** Supervision, Project administration, Funding acquisition.

## Declaration of competing interest

We declare that we have no financial and personal relationships with other people or organizations that can inappropriately influence our work. There is no professional or other personal interest of any nature or kind in any product, service and/or company that could be construed as influencing the position presented in, or the review of the manuscript entitled “Carrier-free Highly Drug-loaded Biomimetic Nanosuspensions Encapsulated by Cancer Cell Membrane Based on Homology and Active Targeting for the Treatment of Glioma”.

## Footnotes

Appendix A Supplementary data to this article can be found online at https://doi.org/10.1016/j.bioactmat.2021.04.027.
