# Supplementary material for: Carrier-free highly drug-loaded biomimetic nanosuspensions encapsulated by cancer cell membrane based on homology and active targeting for the treatment of glioma
Source: Bioact Mater. 2021 May 1;6(12):4402–14. doi: 10.1016/j.bioactmat.2021.04.027 (PMC8111096; doi:10.1016/j.bioactmat.2021.04.027)
Supplement: Multimedia component 1 [file mmc1.doc]

Supporting Information

**Carrier-free Highly Drug-loaded Biomimetic Nanosuspensions Encapsulated by Cancer Cell Membrane Based on Homology and Active Targeting for the Treatment of Glioma**

Yueyue Fan^a,b,1^, Yuexin Cui^b,1^, Wenyan Hao^b,1^, Mengyu Chen^b^, Qianqian Liu^b^, Yuli Wang^b^, Meiyan Yang^b^, Zhiping Li^b^, Wei Gong^b^, Shiyong Song^a,*^,Yang Yang^b,**^, Chunsheng Gao^a,b^

*^a^College of Pharmacy, Henan University, Kaifeng, 475000, PR China*

*^b^State Key Laboratoryof Toxicology and Medical Countermeasures, Beijing Institute of Pharmacology and Toxicology, Beijing, 100850, PR China*

^∗^ Corresponding author.

^∗∗^ Corresponding author.

**Corresponding Author**

Shiyong Song: [pharmsong@outlook.com](mailto:pharmsong@outlook.com); Yang Yang: [amms2013@126.com](mailto:amms2013@126.com);

^1^These authors contributed equally to this work.

**Supplementary Figures**





**Fig. S1.** The MALDI-TOF MS (A) and ^1^H-NMR spectra (B) of DSPE-PEG_2000_-^D^WSW demonstrated the successful synthesis of the targeted ligand. (PTX)NS particle size (C) and zeta potential (F) measurement chart. ^D^WSW-CCM-(PTX) NS particle size (D) and zeta potential (G) measurement chart. Diagrams of particle size (E) and zeta potential (H) of different nanosuspensions. After the cell membrane was coated, the particle size and absolute zeta potential of the nanosuspensions increased.


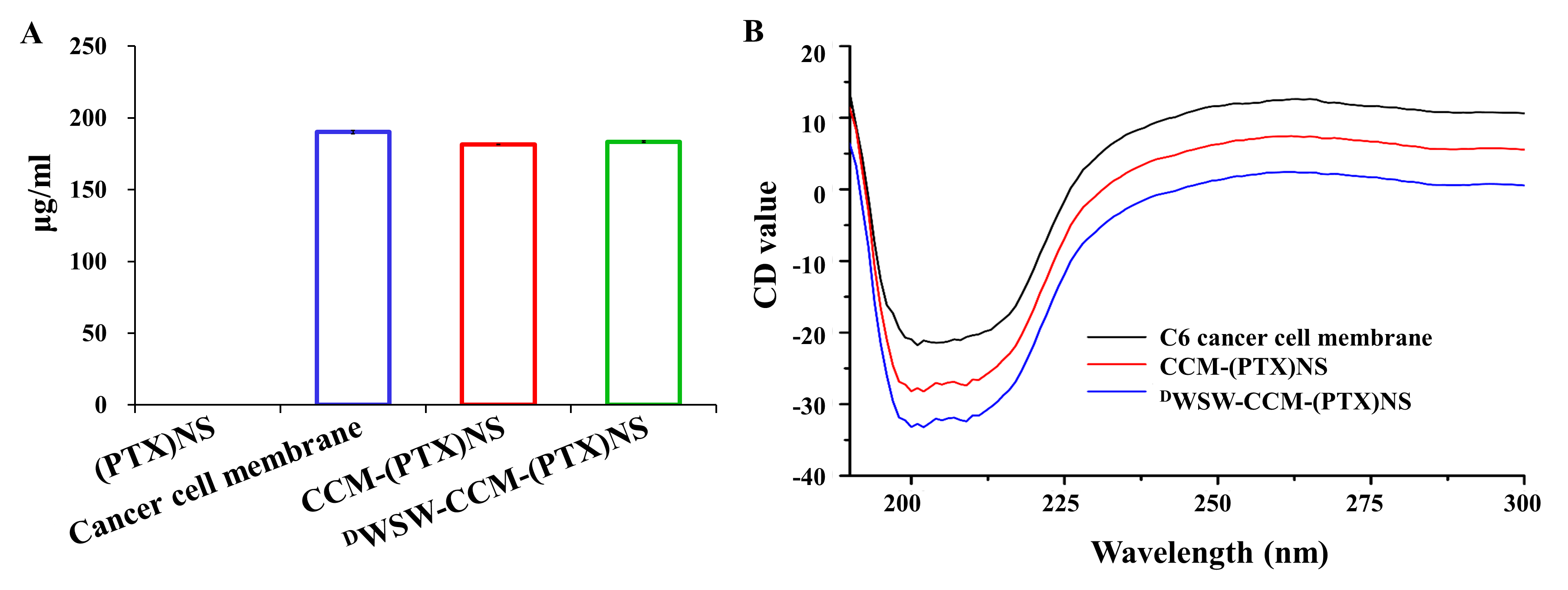


**Fig. S2.** (A) The protein content of cancer cell membranes and biomimetic nanosuspensions was determined using the BCA kit (n=3). (B) The secondary structure of membrane protein was determined by CD. The structure of the protein was unchanged, and the membrane function was retained.


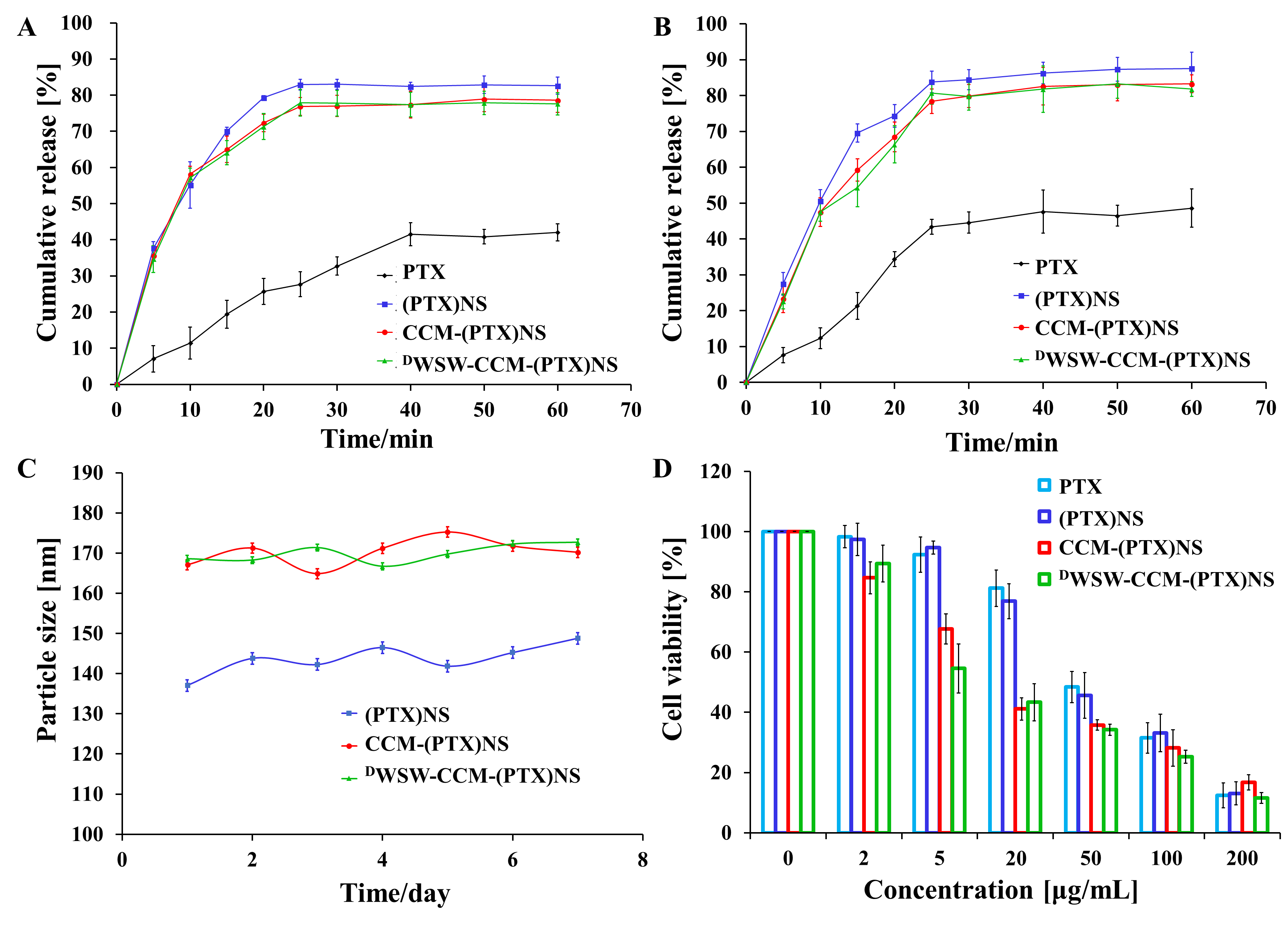


**Fig. S3.** Release curves of PTX, (PTX)NS, CCM-(PTX)NS, and ^D^WSW-CCM-(PTX)NS in PBS (containing 0.5% Tween-80) at pH 7.4 (A) and pH 6.8 (B) (n = 6). (C) The stability of (PTX)NS, CCM-(PTX)NS, and ^D^WSW-CCM-(PTX)NS was maintained at 4 °C for 7 days (n = 3). (D) The inhibitory effect of different drug concentrations on C6 cells showing that the biomimetic nanosuspensions were better (n = 5).


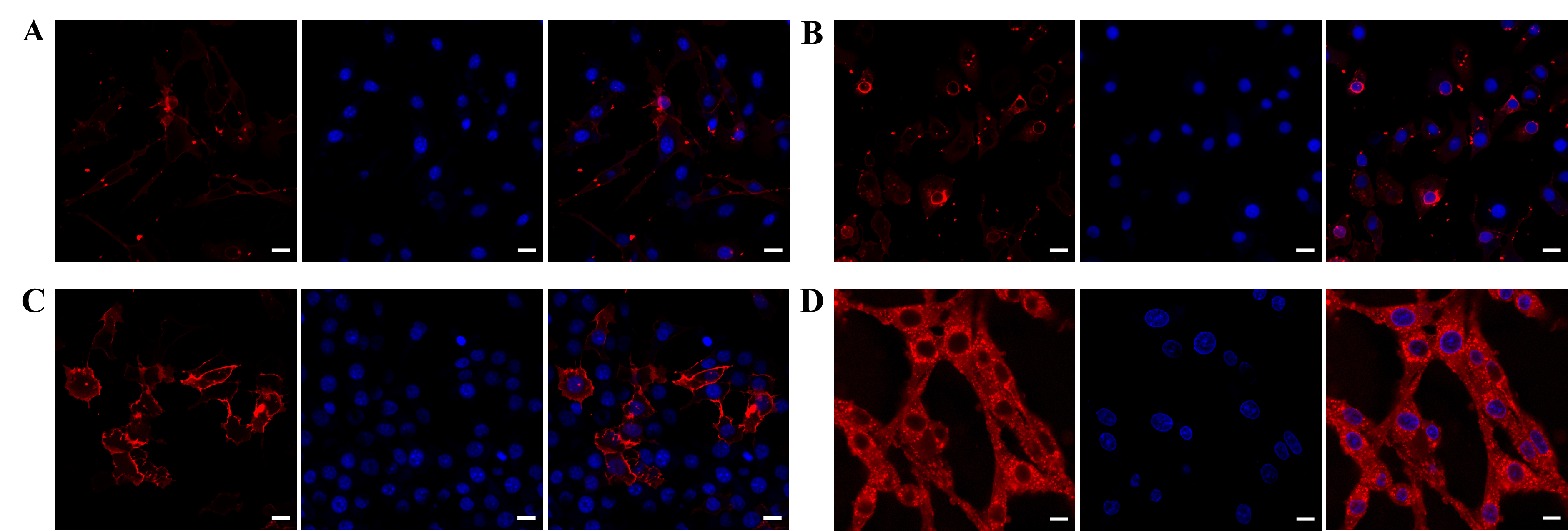


**Fig. S4.** Cell uptake of the biomimetic nanosuspensions by different cancer cells was investigated by CLSM. The intracellular uptake of CCM-(PTX)NS in B16 (A), HepG2 (B), 4T1 (C), and C6 (D). The nuclei were stained with Hoechst 33258 (blue). The CCM-(PTX)NS were labeled with DiI (red) (40x magnification).


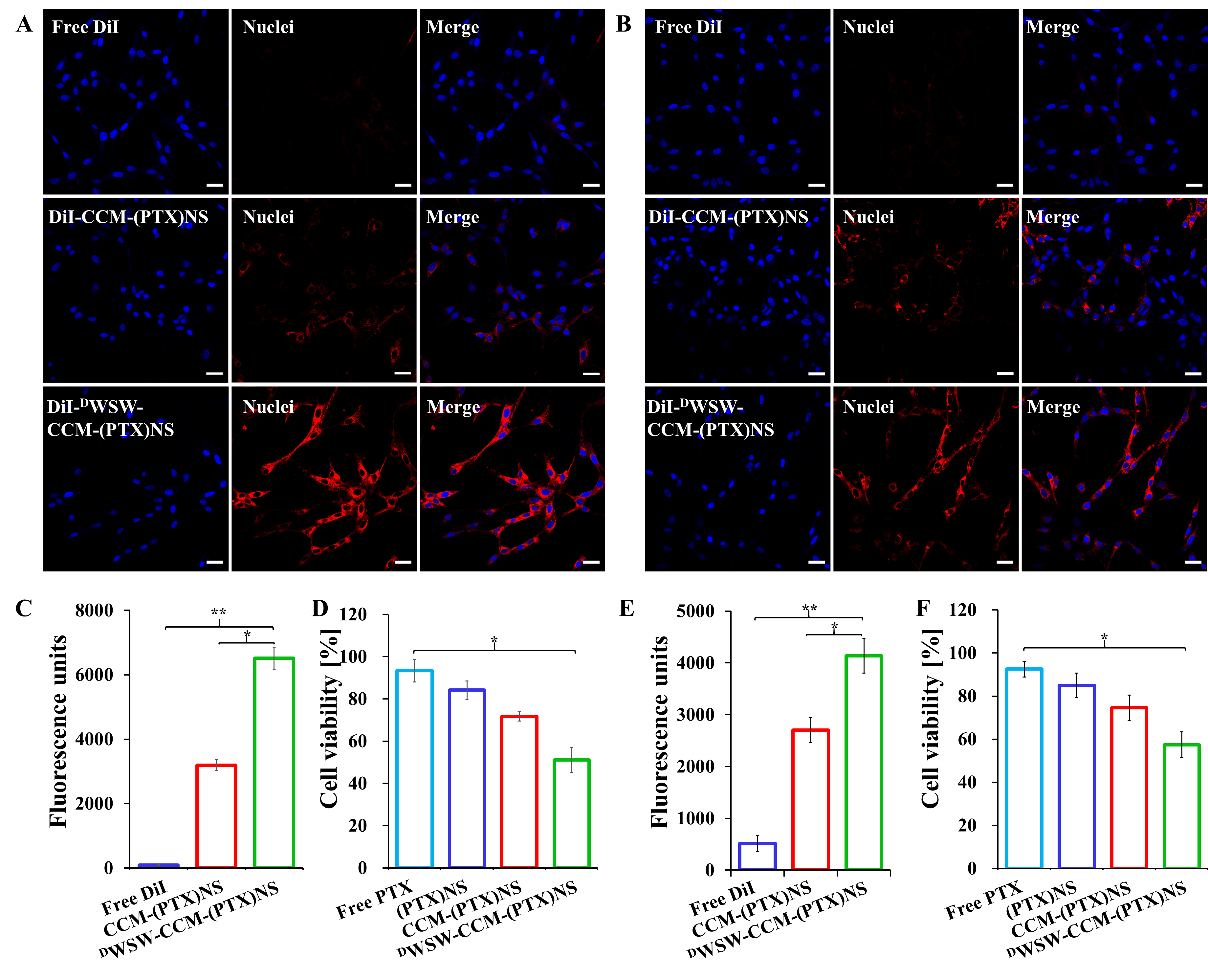


**Fig. S5.** Transcytosis efficiency in BBB and BBTB models. Cell uptake of different preparations by C6 cells in the bEnd.3/C6 *in vitro* BBB model (A) and the HUVEC/C6 BBTB model (B). The fluorescence intensity of the lower liquid and cell viability in the BBB (C and D) and BBTB (E and F) models. (Hoechst 33258: blue, DiI: red; 40x magnification; *p < 0.05, **p < 0.01).


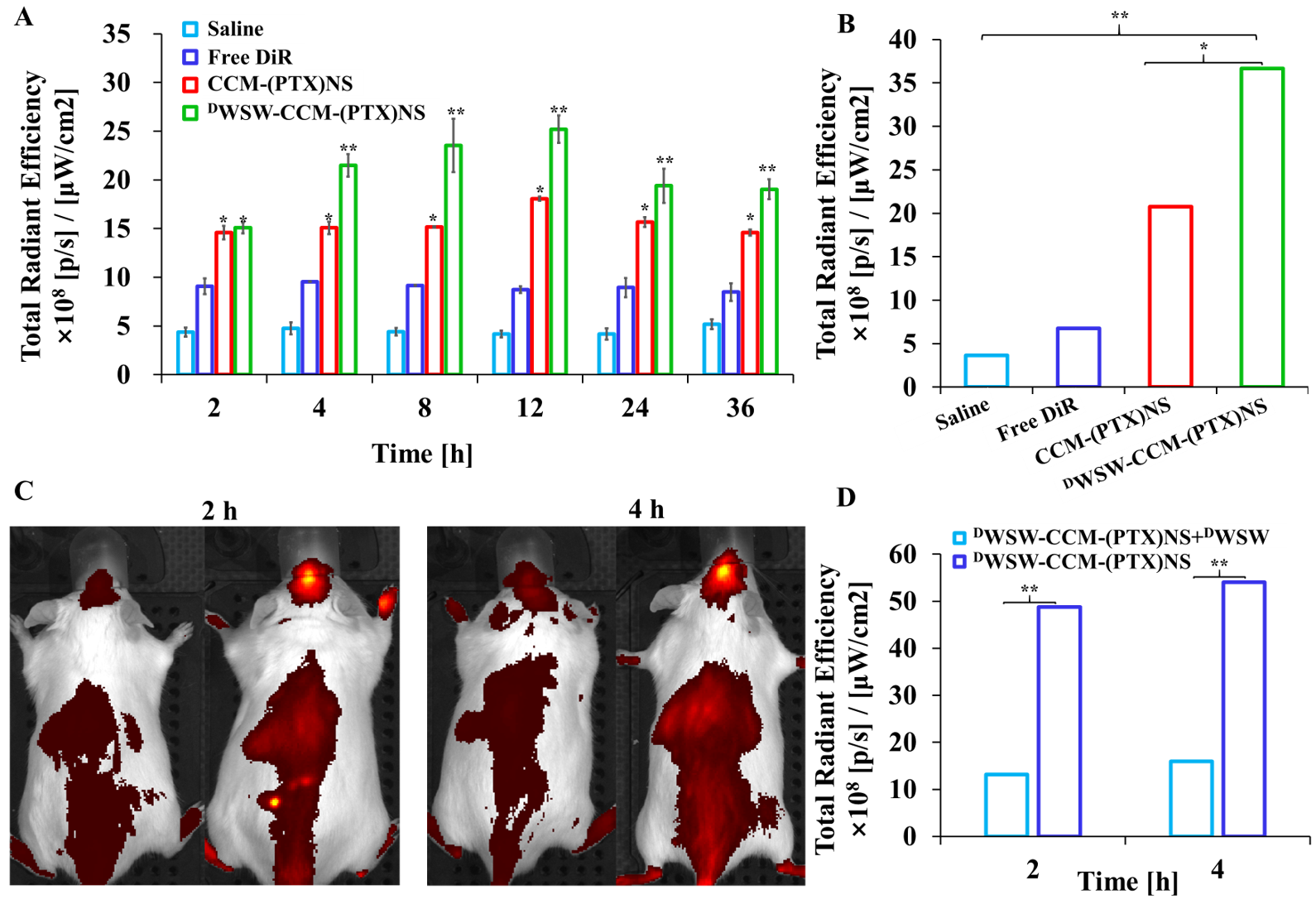


**Fig. S6.** Semi-quantitative ROI results of saline, DiR, CCM-(PTX)NS, and ^D^WSW-CCM-(PTX)NS in glioma-bearing mice (A) and isolated brain tissue (B). Semi-quantitative ROI results (C) and *in vivo* imaging (D) of ^D^WSW-CCM-(PTX)NS + ^D^WSW and ^D^WSW-CCM-(PTX)NS (*p < 0.05, **p < 0.01).


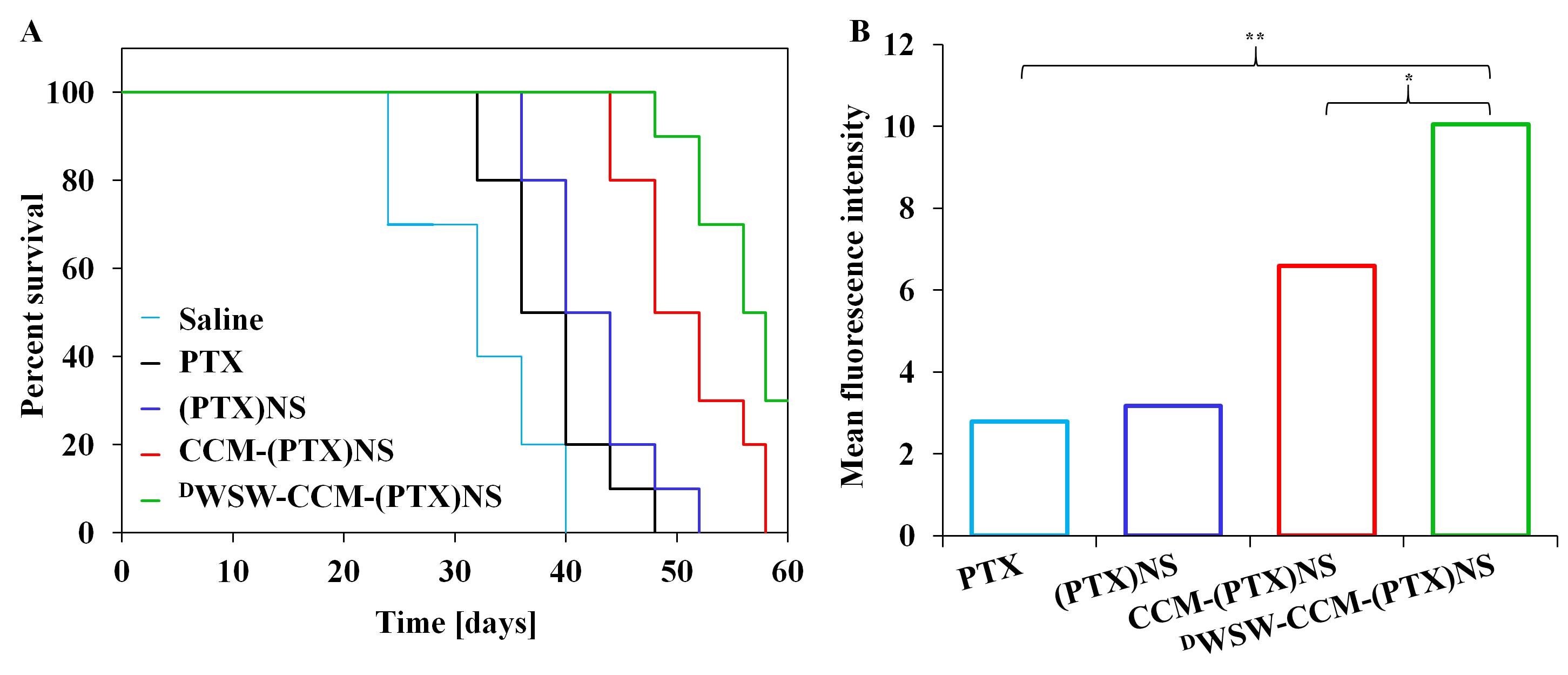


**Fig. S7.** (A) Kaplan-Meier survival curves of mice treated with PTX, (PTX)NS, CCM-(PTX)NS, and ^D^WSW-CCM-(PTX)NS. (B) ImageJ was used to quantify TUNEL staining (*p < 0.05, **p < 0.01).
